# Supplementary figures and images for: Variants of MIRNA146A rs2910164 and MIRNA499 rs3746444 are associated with the development of cutaneous leishmaniasis caused by Leishmania guyanensis and with plasma chemokine IL-8
Source: PLoS Negl Trop Dis. 2021 Sep 20;15(9):e0009795. doi: 10.1371/journal.pntd.0009795 (PMC8483412; doi:10.1371/journal.pntd.0009795)

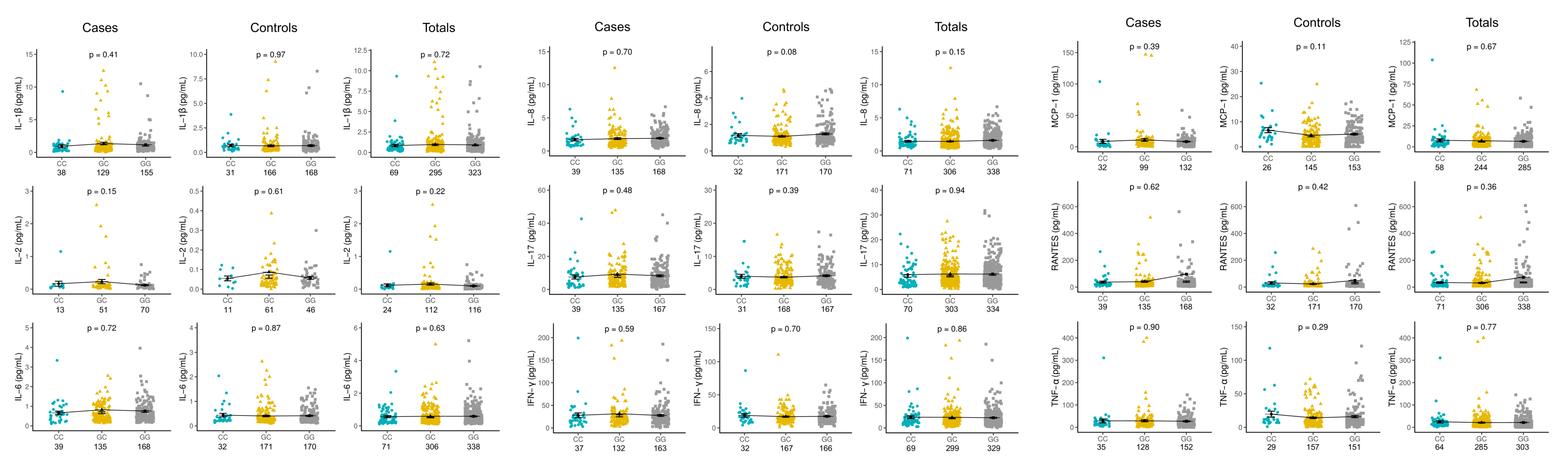

Supplement: S1 Fig — (TIFF) [file pntd.0009795.s001.tiff]

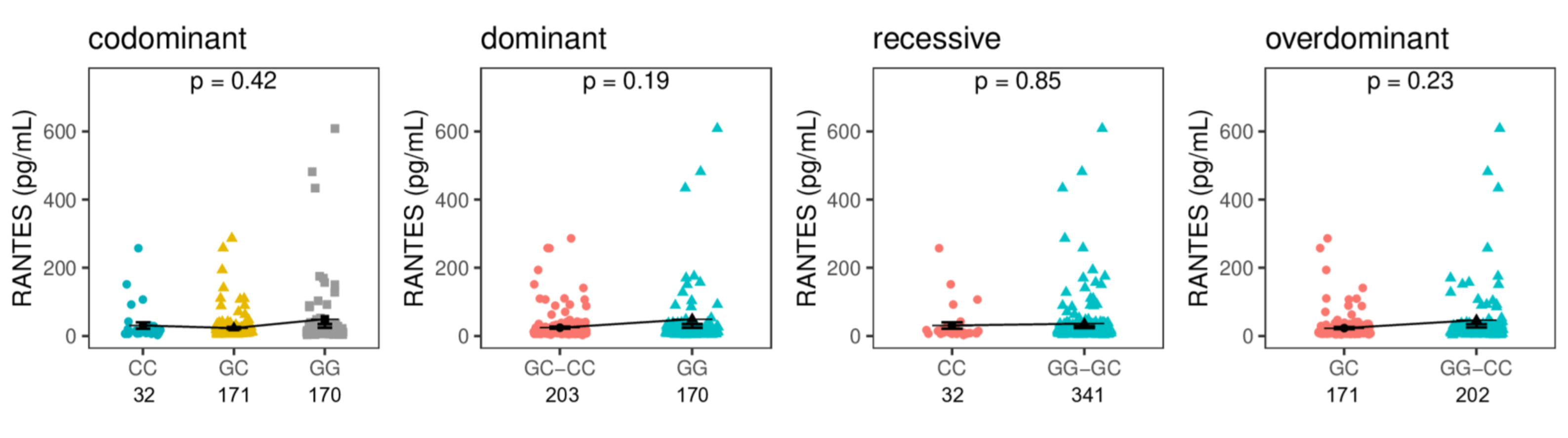

Supplement: S2 Fig — (TIFF) [file pntd.0009795.s002.tiff]

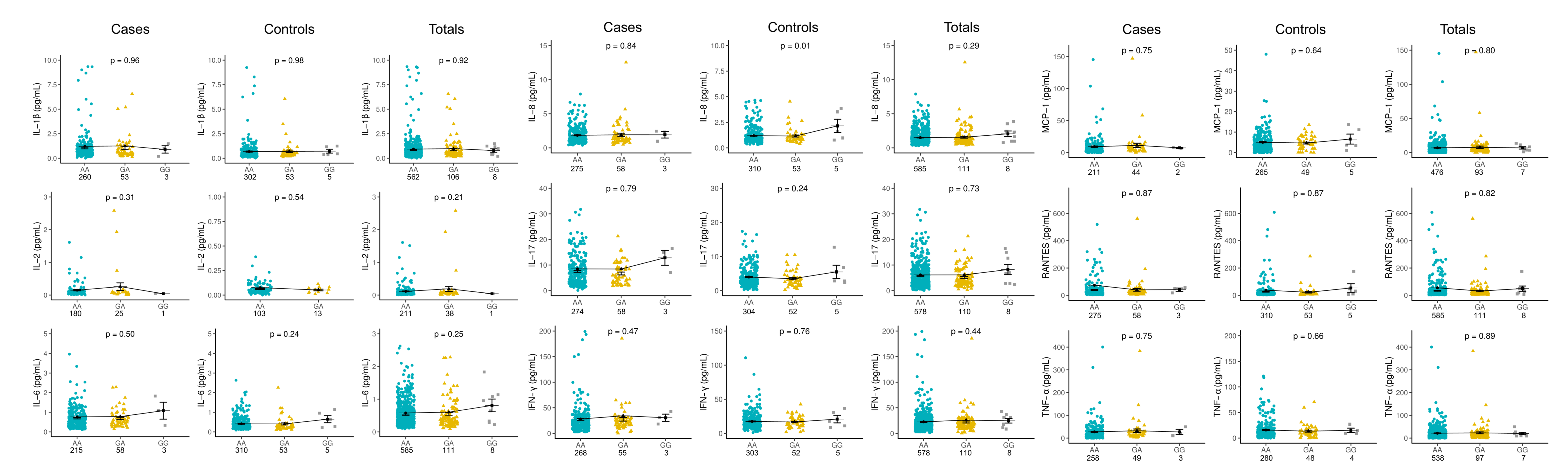

Supplement: S3 Fig — (TIFF) [file pntd.0009795.s003.tiff]

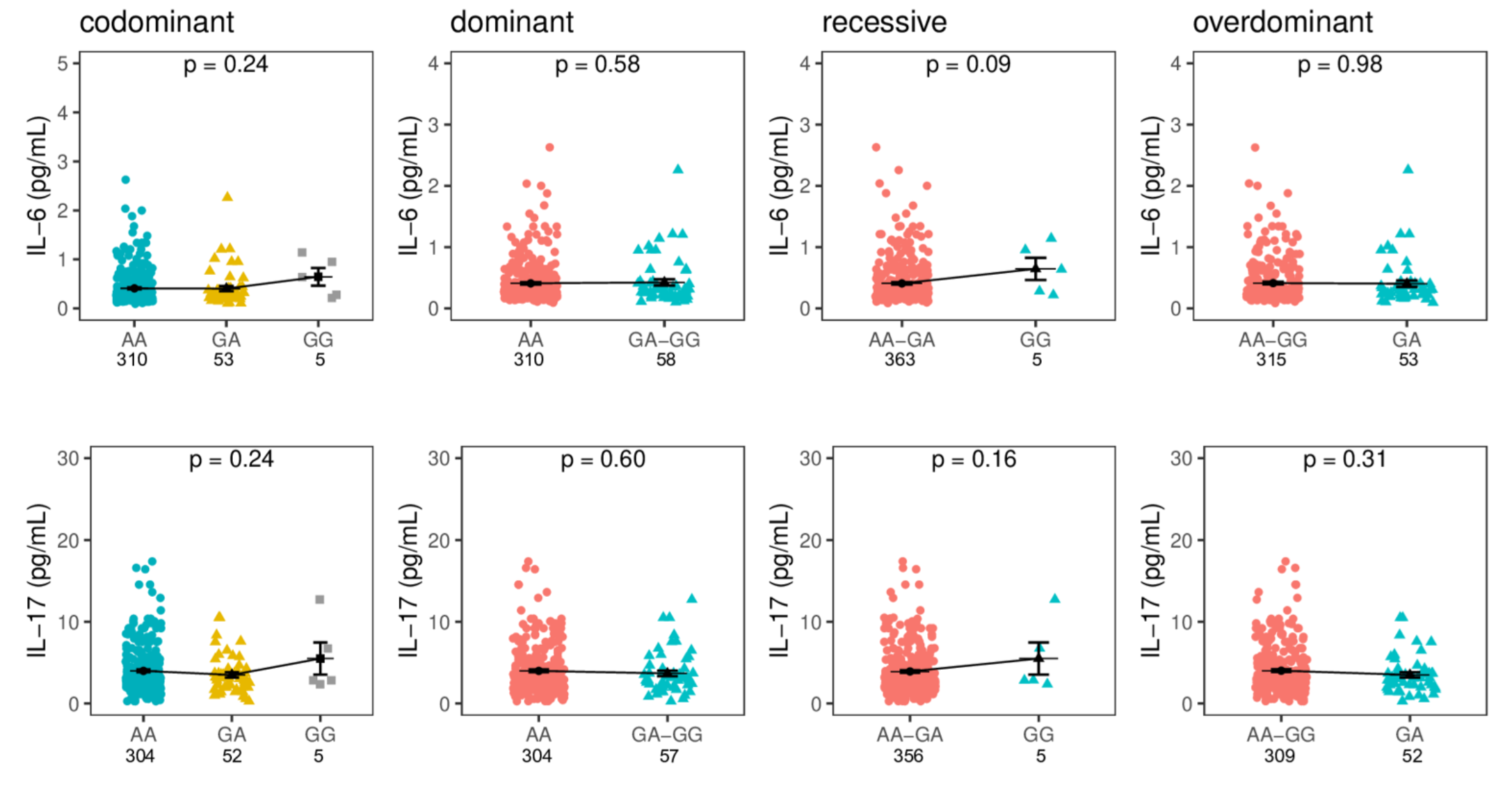

Supplement: S4 Fig — (TIFF) [file pntd.0009795.s004.tiff]

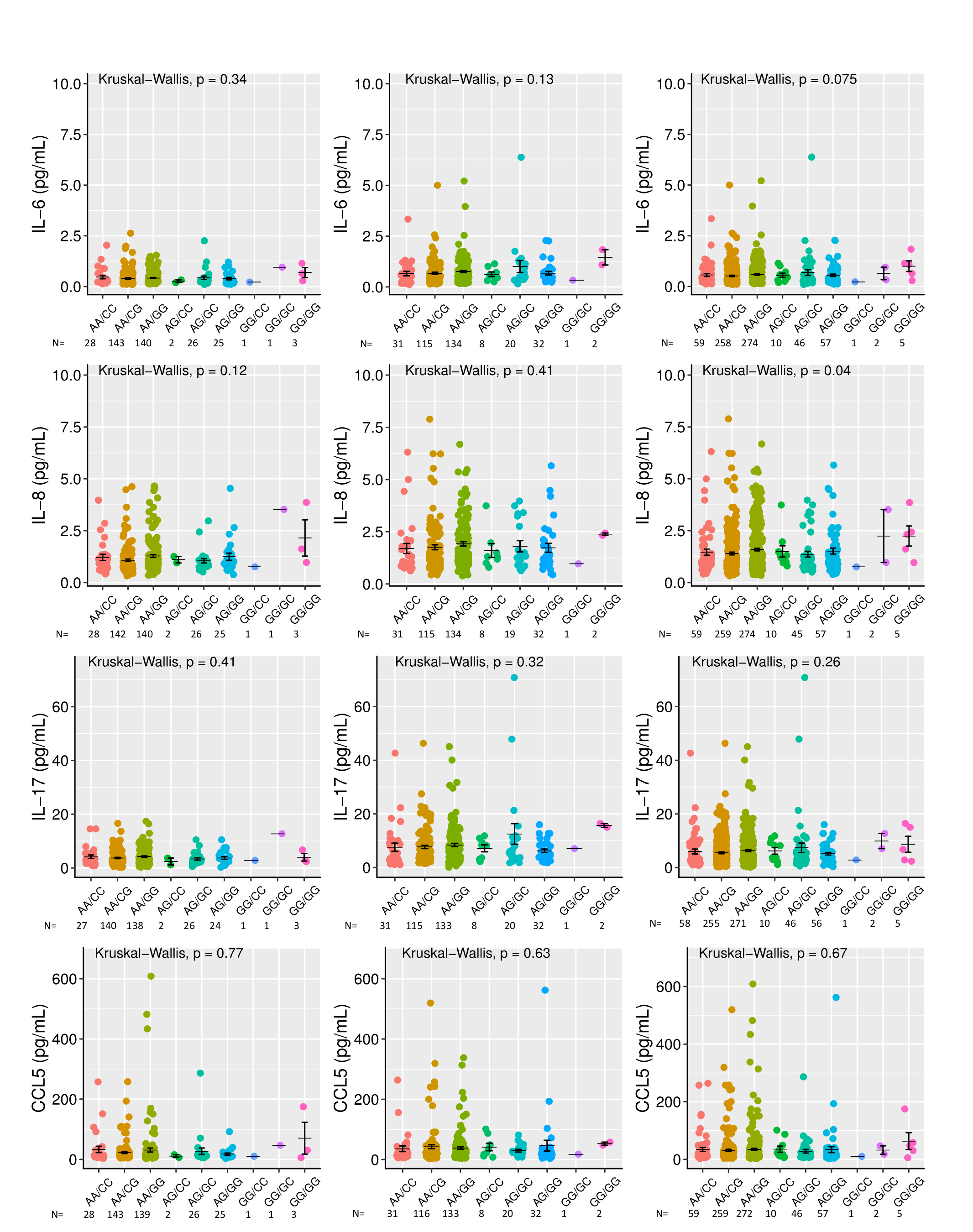

Supplement: S5 Fig — (TIFF) [file pntd.0009795.s005.tiff]
