# Supplementary material for: Variants of MIRNA146A rs2910164 and MIRNA499 rs3746444 are associated with the development of cutaneous leishmaniasis caused by Leishmania guyanensis and with plasma chemokine IL-8
Source: PLoS Negl Trop Dis. 2021 Sep 20;15(9):e0009795. doi: 10.1371/journal.pntd.0009795 (PMC8483412; doi:10.1371/journal.pntd.0009795)
Supplement: S1 Table — (DOCX) [file pntd.0009795.s006.docx]

| Primer sequence 5’-3’ | Polymerase Chain Reaction protocol | Restriction enzymes | Allele; length in base pairs |
| --- | --- | --- | --- |
| *MIR146A* rs2910164  F: CATGGGTTGTGTCAGTGTCAGAACT  R: GCCTTCTGTCTCCAGTCTTCC | 95 °C for 5 min; 40 cycles (95 °C for 15 secs; 58 °C for 15 secs and 72 °C for 30 secs); 72 °C for 7 min. | HpyCH4 III | Allele G: 121+25  Allele C :146 |
| *MIR499A rs3746444*  F:AGTGATGTTTAACTCCTCTCCGCGTGA  R: CACTTCCCTGCCAAATCCCCGTCC | 95 °C for 5 min; 40 cycles (95 °C for 15 secs; 60 °C for 15 secs and 72 °C for 30 secs); 72 °C for 7 min. | HpyCH4 IV | Allele G: 112+28  Allele A:140 |
