# Supplementary material for: Variants of MIRNA146A rs2910164 and MIRNA499 rs3746444 are associated with the development of cutaneous leishmaniasis caused by Leishmania guyanensis and with plasma chemokine IL-8
Source: PLoS Negl Trop Dis. 2021 Sep 20;15(9):e0009795. doi: 10.1371/journal.pntd.0009795 (PMC8483412; doi:10.1371/journal.pntd.0009795)
Supplement: S2 Table — (DOCX) [file pntd.0009795.s007.docx]

| *MIR146A* | IL-1β | | | IL-2 | | | IL-6 | | | IL-8 | | | IL-17 | | | IFN-γ | | | MCP-1 | | | RANTES | | | TNF-α | | | |
| --- | --- | --- | --- | --- | --- | --- | --- | --- | --- | --- | --- | --- | --- | --- | --- | --- | --- | --- | --- | --- | --- | --- | --- | --- | --- | --- | --- | --- |
| Inheritance model | **n** | **me** | **se** | **n** | **me** | **se** | **n** | **me** | **se** | **n** | **me** | **se** | **n** | **me** | **se** | **n** | **me** | **se** | **n** | **me** | **se** | **n** | **me** | **se** | **n** | **me** | **se** |  |
| G/G  C/G  C/C | 323  295  69 | 0.92  0.97  0.83 | 0.08  0.09  0.14 | 116  112  24 | 0.09  0.15  0.11 | 0.01  0.03  0.04 | 338  306  71 | 0.58  0.54  0.56 | 0.02  0.03  0.06 | 338  306  71 | 1.60  1.43  1.47 | 0.05  0.06  0.12 | 334  303  70 | 6.17  6.22  5.91 | 0.30  0.59  0.76 | 329  299  69 | 22.56  23.55  23.77 | 1.03  1.95  3.19 | 285  244  58 | 6.57  7.27  7.74 | 0.38  0.95  1.81 | 338  306  71 | 73.01  31.13  34.02 | 30.14  3.01  5.93 | 303  285  64 | 21.48  21.02  24.48 | 1.30  2.17  5.10 |  |
| G/G  G/C-C/C | 323  364 | 0.92  0.94 | 0.08  0.08 | 116  136 | 0.09  0.15 | 0.01  0.02 | 338  377 | 0.58  0.54 | 0.02  0.03 | 338  377 | 1.60  1.43 | 0.05  0.06 | 334  373 | 6.17  6.16 | 0.30  0.50 | 329  368 | 22.56  23.59 | 1.03  1.69 | 285  302 | 6.57  7.36 | 0.38  0.84 | 338  377 | 73.01  31.68 | 30.14  2.68 | 303  349 | 21.48  21.65 | 1.30  2.00 |  |
| G/G-G/C  C/C | 618  69 | 0.94  0.83 | 0.06  0.14 | 228  24 | 0.12  0.11 | 0.01  0.04 | 644  71 | 0.56  0.56 | 0.02  0.06 | 644  71 | 1.52  1.47 | 0.04  0.12 | 637  70 | 6.19  5.91 | 0.32  0.76 | 628  69 | 23.03  23.77 | 1.07  3.19 | 529  58 | 6.89  7.74 | 0.48  1.81 | 644  71 | 53.11  34.02 | 15.89  5.93 | 588  64 | 21.26  24.48 | 1.24  5.10 |  |
| G/G-C/C  G/C | 392  295 | 0.90  0.97 | 0.07  0.09 | 140  112 | 0.09  0.15 | 0.01  0.03 | 409  306 | 0.58  0.54 | 0.02  0.03 | 409  306 | 1.57  1.43 | 0.05  0.06 | 404  303 | 6.12  6.22 | 0.28  0.59 | 398  299 | 22.77  23.55 | 1.01  1.95 | 343  244 | 6.77  7.27 | 0.43  0.95 | 409  306 | 66.24  31.13 | 24.93  3.01 | 367  285 | 22.00  21.02 | 1.39  2.17 |  |
| *MIR499A* |  | | |  | | |  | | |  | | |  | | |  | | |  | | |  | | |  | | |  |
| A/A  A/G  G/G | 562  106  8 | 0.93  0.97  0.78 | 0.06  0.15  0.17 | 211  38  1 | 0.11  0.19  0.04 | 0.01  0.08  0.00 | 585  111  8 | 0.55  0.60  0.80 | 0.02  0.06  0.19 | 585  111  8 | 1.49  1.56  2.05 | 0.04  0.13  0.41 | 578  110  8 | 6.14  6.08  8.23 | 0.33  0.79  2.02 | 571  107  8 | 22.52  26.13  24.50 | 0.95  4.23  4.39 | 476  93  7 | 6.86  7.70  6.79 | 0.48  1.67  1.69 | 585  111  8 | 55.30  31.61  48.97 | 14.47  5.91  19.19 | 538  97  7 | 21.43  23.05  20.26 | 1.27  4.28  5.46 |  |
| A/A  A/G-G/G | 562  114 | 0.93  0.96 | 0.06  0.14 | 211  39 | 0.11  0.19 | 0.01  0.08 | 585  119 | 0.55  0.62 | 0.02  0.06 | 585  119 | 1.49  1.59 | 0.04  0.13 | 578  118 | 6.14  6.23 | 0.33  0.75 | 571  115 | 22.52  26.02 | 0.95  3.94 | 476  100 | 6.86  7.64 | 0.48  1.56 | 585  119 | 55.30  32.78 | 17.47  5.66 | 538  104 | 21.43  22.87 | 1.27  4.01 |  |
| A/A-A/G  G/G | 668  8 | 0.94  0.78 | 0.06  0.17 | 249  1 | 0.12  0.04 | 0.01  0.00 | 696  8 | 0.56  0.80 | 0.02  0.19 | 696  8 | 1.50  2.05 | 0.04  0.41 | 688  8 | 6.13  8.23 | 0.30  2.02 | 678  8 | 23.09  24.50 | 1.04  4.39 | 596  7 | 6.99  6.79 | 0.48  1.69 | 696  8 | 51.52  48.97 | 14.71  19.19 | 635  7 | 21.68  20.26 | 1.26  5.46 |  |
| A/A-G/G  A/G | 570  106 | 0.93  0.97 | 0.06  0.15 | 212  38 | 0.11  0.19 | 0.01  0.08 | 593  111 | 0.55  0.60 | 0.02  0.06 | 593  111 | 1.50  1.56 | 0.04  0.13 | 586  110 | 6.16  6.08 | 0.32  0.79 | 579  107 | 22.55  26.13 | 0.94  4.23 | 483  93 | 6.85  7.70 | 0.47  1.67 | 593  11 | 55.22  31.61 | 17.24  5.91 | 545  97 | 21.42  23.05 | 1.25  4.28 |  |
| n: sample size; me: mean (pg/mL); se: standard error | | | | | | | | | | | | | | | | | | | | | | | | | | | |  |
