# Supplementary material for: Variants of MIRNA146A rs2910164 and MIRNA499 rs3746444 are associated with the development of cutaneous leishmaniasis caused by Leishmania guyanensis and with plasma chemokine IL-8
Source: PLoS Negl Trop Dis. 2021 Sep 20;15(9):e0009795. doi: 10.1371/journal.pntd.0009795 (PMC8483412; doi:10.1371/journal.pntd.0009795)
Supplement: S3 Table — (DOCX) [file pntd.0009795.s008.docx]

| **Combined genotypes** | **Patients with CL** | | | **Healthy controls** | | |
| --- | --- | --- | --- | --- | --- | --- |
| **rs3746444/**  **rs2910164** | **Males**  **n=597** | **Females**  **n=198** | **Total**  **n=795** | **Males**  **n=578** | **Females**  **n=268** | **Total**  **n=846** |
| AA/GG | 209 (35) | 74 (37) | 283 (36) | 226 (39) | 91 (34) | 317 (37) |
| AA/GC | 194 (32) | 76 (38) | 270 (34) | 210 (36) | 106 (40) | 316 (37) |
| AA/CC | 63 (10) | 16 (8) | 79 (10) | 47 (8) | 21 (8) | 68 (8) |
| AG/GG | 61 (10) | 9 (1) | 70 (9) | 43 (7) | 23 (9) | 66 (8) |
| AG/GC | 43 (7) | 17 (8) | 60 (7) | 38 (6) | 21 (8) | 59 (7) |
| AG/CC | 16 (3) | 2 (1) | 18 (2) | 7 (1,2) | 3 (1,1) | 10 (1) |
| GG/GG | 6 (1) | 3 (1,5) | 9 (1) | 4 (0,7) | 1 (0,3) | 5 (0,5) |
| GG/GC | 3 (0,5) | 0 | 3 (0,4) | 2 (0,3) | 1 (0,3) | 3 (0,3) |
| GG/CC | 2 (0,3) | 1 (0,5) | 3 (0,4) | 1 ( 0,1) | 1 (0,3) | 2 (0,2) |

**MALES**

AA/GG vs AA/GC + AG/GG OR [CI] P-value

CL 209 194 61 1.1 [0.84-1.4] 0.25

HC 226 210 43

AA/GG vs AA/CC + AG/GC + GG/GG OR [CI] P-value

CL 209 63 43 6 1.4 [1.0-1.9] 0.035

HC 226 47 38 4

AA/GG vs GG/CC + AG/CC + GG/GC OR [CI] P-value

CL 209 2 16 3 2.3 [1.0-4.7] 0.017

HC 226 1 7 2

**FEMALES**

AA/GG vs AA/GC + AG/GG OR [CI] P-value

CL 74 76 9 0.8 [0.5-1.2] 0.16

HC 91 106 23

AA/GG vs AA/CC + AG/GC + GG/GG OR [CI] P-value

CL 74 16 17 3 1.0 [0.6-1.7] 0.46

HC 91 21 21 1

AA/GG vs GG/CC + AG/CC + GG/GC OR [CI] P-value

CL 74 1 2 0 0.7 [0.2-2.9] 0.36

HC 91 1 3 1

**TOTAL**

AA/GG vs AA/GC + AG/GG OR [CI] P-value

CL 283 270 70 1.0 [0.8-1.2] 0.49

HC 317 316 66

AA/GG vs AA/CC + AG/GC + GG/GG OR [CI] P-value

CL 283 79 60 9 1.3 [0.9-1.7] 0.06

HC 317 68 59 5

AA/GG vs GG/CC + AG/CC + GG/GC OR [CI] P-value

CL 283 3 18 3 1.8 [0.9-3.4] 0.04

HC 317 2 10 3
