# Supplementary material for: Variants of MIRNA146A rs2910164 and MIRNA499 rs3746444 are associated with the development of cutaneous leishmaniasis caused by Leishmania guyanensis and with plasma chemokine IL-8
Source: PLoS Negl Trop Dis. 2021 Sep 20;15(9):e0009795. doi: 10.1371/journal.pntd.0009795 (PMC8483412; doi:10.1371/journal.pntd.0009795)
Supplement: Attachment — Submitted filename: Response to reviewers.docx [file pntd.0009795.s009.docx]

REPLY TO THE REVIEWERS

**Reviewer 1**

Reviewer #1: The objective of performing the polymorphism analysis is clear; the cytokine measurements are too indirect. The population is well described, although the sample is highly biased for sex. Some analysis accounted for that but not all. there is no information regarding sample size calculation. No concerns related to ethical requirements.

***We agree with your comment. We stratified our sample by gender since there are more women in our healthy controls group. As far as the sample size calculation, we have applied a strictly rigorous method as referred in our previous article (DOI: 10.1093/infdis/jiab320).***

***The effective sample size was calculated for case-control discrete trait online using the Genetic Power calculator developed at Harvard University (***[***http://pngu.mgh.harvard.edu/~purcell/gpe***](http://pngu.mgh.harvard.edu/~purcell/gpe)***) under the assumptions of a minor allele frequency of 5%, disease prevalence of 5%, complete***

***linkage disequilibrium 1 between marker and trait, case-control ratio 1, and 5% type 1 error rates with an odds ratio of 1.5 and 2.0 for heterozygosity and homozygosity, respectively. For 80% power, the genetic allelic model generated a sample size por 789 for cases and 789 for controls****.*

Reviewer #1: Conclusions are partially supported by the data, as the authors presented a lengthy and highly speculative discussion, which should be modified. Some limitations are presented in the discussion, but it would be beneficial to list them separately, rather than embedded in the discussion.

***We have reduced the discussion section and presented a paragraph about the limitations of the study*.**

Reviewer #1: - Please see nomenclature for chemokines (RANTES/CCL5 and MCP1/CCL2 no longer used).

***Corrected as CCL5 and CCL2 respectively****.*

Reviewer #1: The manuscript by Mesquita et al. evaluated the association between genetic variants of miR146-a and miR499a and cutaneous leishmaniasis (CL) caused by L. guyanensis. They evaluated the frequency of the variants rs2910164 and rs3746444 in 850 cases and 891 controls by RFLP. In addition, the authors evaluated the expression of selected cytokines using Bioplex. The data showed that CC genotype for the rs2910164 is slightly associated with CL, while the GG genotype for rs3746444 is associated with a 75% higher risk of developing CL. The paper also shows the expression of selected inflammatory cytokines and chemokines, attempting to correlate miRNA variants and cytokine levels.

The paper deals with an important subject, which is to find markers of susceptibility to Leishmania infection, a devastating disease. I feel that the manuscript should focus on the association of the variants with disease, as the experiments with cytokine levels are too indirect. The results can be combined as a short report. Please see some specific comments below:

- The authors adjusted the statistical analysis for sex and age in table 1 (Padj column). This correction did not return statistically significant differences. However, data presented in table 2, where they segregated by sex (not considering age) showed statistically significant associations.
Does this suggest that age, in association with sex, is influencing the results? How do the authors explain that?

***We have included in Table 2. P values are adjusted for sex and showed that age does not have influence on the association.***

- Does the combined genotype analysis (for example: occurrence of CC genotype for rs2910164 together with GG genotype for rs3746444) show a higher risk of disease development?

***We showed that genetics combinations do increase the risk as shown in supplementary Table 3.***

- Cytokine expression is influenced by several parameters (such as immunological, environmental, genetic, and epigenetic). However, while these miRNAs have been associated with consequences in cytokine expression due to their action in signaling molecules, it is not clear that the variants for these miRNAs are functional regarding the expression of the miRNA itself.
Have the authors measured the levels of miR146-a and miR499a in individuals with distinct genotypes? This would help make the argument of the functional activity of the variants and would help associate with cytokine expression.

***Although the measurement of plasma cytokines is an indirect method compared to the quantification of the miRNA, we have chosen only cytokines where both miRNAs may have an influence. We believe that measuring plasma cytokines may reflect natural context of an individual since it is not under experimental control as in vitro studies. Furthermore, we all know very well that cytokine expression is influenced by several parameters (such as immunological, environmental, genetic, and epigenetic).***

***We did not measure the levels of miRNA because several groups have done this. We did cite in the discussion section:***

***Line 459: “The MIR146A is in the cytokine cluster 5q31 and the MIR146A rs2910164 is situated in the stem region of the pre-miR-146a. The change of guanine:uracil pair (G:U) to cytosine:uracil (C:U) in the stem structure affects the production of mature miR146-a [25,66]. The G-allele is reported to be associated with high expression levels of mature miR146-a while the C-allele with low expression [25, 67]. Other study cited that the C allele is correlated with higher miR146-a and lower TNFα expression from nerve biopsies of leprosy patients [42]. High expression of miR146-a was also observed in specimens of healthy and tumour tissue from patients with gastric cancer bearing genotype CC compared to GG [68]. In Lupus, CC genotypes was correlated with high miR146-a expression [69].***

***Line 490: “MIR499A rs3746444 genotype GG has been suggested to correlate with high expression of miR499-a.” Ref. 83.***

- The fact that the variants are associated with expression of some but not all cytokines that are related to the molecules inhibited by the miRNA suggests that factors other than the variants influence cytokine expression. In addition, as mentioned above, the authors do not show an association between the variant and the expression of the miRNA. Thus, the cytokine data represents too far of an extrapolation of between the variant for the miRNA and its functional activity and because of that it should be removed.

***We agree to disagree. Although we did not measure miRNA, we related these variants with most the cytokines that been described to be influenced by both miRNAs. We think that these correlations may be helpful for other group of researchers. We did not only show the one showing significance by genotypes.***

- Discussion is too long and speculative.

***We have done our best to reduce the discussion.***

**Reviewer 2**

Reviewer #2: The methods is in consonante with goal proposed in the Manuscript, supporting the objetives. The statistical analysis was adequate.

A minor point can be included in the text:

What is the volume of PCR mix and Buffer? And the volume of enzymatic digestion mix?

***We have included in the text.***

Reviewer #2: Tables and Figures are clearly and showed that an analysis of SNPs MIR146A rs2910164 and MIR499A rs3746444 showed a prevalence of MIR146A rs2910164 CC genotype and MIR499A rs3746444 AA genotype, elevating the risk to develop CL. In MIR146A rs2910164 G allele presented higher levels of IL-8 and RANTES in CL plasma samples. The, MIR499A rs3746444 G allele presented higher levels of IL-8, IL-6 and IL-17.

This information open some questions:

- Did you analyze the prevalence of both MIR146A rs2910164 CC and MIR499A rs3746444 AA genotypes in the same CL patients? These correlates?

***We studied the same patients and controls for both variants. As requested, we have performed genetics combinations as shown in supplementary table 3 and correlate with plasma cytokines IL-8, IL-6 and IL-17 and CCL5. We have included in the result sections line 336-343; 409-411 supplementary figure 5.***

***“Genetic combinations of both MIR146A rs2910164 and MIR499A rs3746444 genotypes***

***The frequencies of the combined genotypes are shown in Supplementary Table 3. Male individuals carrying at least one susceptibility allele (MIR146A rs2910164C or MIR499A rs3746444G) revealed an OR of 1.1 [0.8-1.4] in comparison to individuals with two alleles (1.4 95% CI [1.0-1.9]; p=0.035) or three or four alleles (2.3 95% CI [1.0-4.7]; p=0.017). Notably, these associations were not observed among female individuals.”***

-    In MIR146A rs2910164 G allele presented higher levels of IL-8 and RANTES in CL plasma samples. The, MIR499A rs3746444 G allele presented higher levels of IL-8, IL-6 and IL-17. Have some correlation of presence of both rs2910164 G allele and rs3746444 G allele and cytokines in the same CL patients?

***Thank you for reminding us to do genetic combinations of both variants. We have included this analysis in the text included a supplementary figure 5. Lines 409-411…***

Reviewer #2: Authors studied the SNPs in miR1461 and miR499 in Leishmania guyanensis CL patients. The manuscript Bring a new View point to understand the function of the SNP in the

miRNAs, a molecules that was found impacting in the host-parasite interaction, correlating with suceptibility or resistance to infection.

***Thank you for appreciating the study****.*

Reviewer #2: I consider the work address a higher relevance to understand how the host-genetic interfere in the post-transcriptional regulation of gene expression and consequences to inflammation. I recommend a minor revision.

Minor points:

Abstract: include the main conclusion in the final of abstract.

***Corrected***

In the introduction section:

author can cite the original works that describe the miRNA function, as Refs: Baltimore et al., 2008; Bartel, 2004; Bartel, 2009.

***Included***

The work from Geraci and others can be used to explain the impact of miRNAs in host susceptibility to infection, as REF: Lemaire et al., 2013; Geraci et al., 2015; Muxel et al., 2017,2018; Acuna et al., 2020; Tiwari et al., 2017, Nunes et al., 2018, Kumar et al, 2018; Das et al., 2020; Souza et al.,2021 and others

***We have cited some of the reference*.**

- Are the miR-146a and miR-499 upregulated in Leishmania-infected human/mouse host to support the evaluation of miRNA polymorphisms? Inlcude some information in the introduction and discussion sections. As REFs:

- Kumar V, Kumar A, Das S, et al. Leishmania donovani Activates Hypoxia Inducible Factor-1α and miR-210 for Survival in Macrophages by Downregulation of NF-κB Mediated Pro-inflammatory Immune Response. Front Microbiol. 2018;9:385. Published 2018 Mar 8. doi:10.3389/fmicb.2018.00385 ;
- Das S, Mukherjee S, Ali N (2021) Super enhancer-mediated transcription of miR146a-5p drives M2 polarization during Leishmania donovani infection. PLoS Pathog 17(2): e1009343. <https://doi.org/10.1371/journal.ppat.1009343>
-Diotallevi A, De Santi M, Buffi G, Ceccarelli M, Vitale F, Galluzzi L and Magnani M (2018) Leishmania Infection Induces MicroRNA hsa-miR-346 in Human Cell Line-Derived Macrophages. Front. Microbiol. 9:1019. doi: 10.3389/fmicb.2018.01019

***We included some of the references****.*

**Reviewer 3**

Reviewer #3: Major comments:

1. Correction for multiple testing is strictly necessary for the cytokine analysis, since the Methods sections mentions 8 different cytokines/chemokines were measured: “Cytokine assay of IL-1β, TNF-α, IL-2, IL-6, IL-8, IL-17, IFN-γ, MCP-1 and RANTES in the plasma were measured using the multiplex cytokine commercial kit Bio-PlexProTM Human Cytokine Grp I Panel 27-Plex (Bio-Rad, USA).” Bonferroni correction (all p-values multiplied by 8) might be too harsh, but at least FDR correction (Benjamini-Hochberg or similar) should be applied to all comparisons. In fact, this is a minimal approach, since for each association 4 genetic models were tested in 3 situations (cases, controls and total), correcting for all these would result in no significant associations in the entire study.

However, it seems 27 different chemokines/cytokines were actually quantified with the Bioplex kit, according to the manufacturer:

•    FGF basic
•    Eotaxin
•    G-CSF
•    GM-CSF
•    IFN-γ
•    IL-1β
•    IL-1ra

•    IL-2
•    IL-4
•    IL-5
•    IL-6
•    IL-7
•    IL-8
•    IL-9    •    IL-10
•    IL-12 (p70)
•    IL-13
•    IL-15
•    IL-17A
•    IP-10
•    MCP-1 (MCAF)    •    MIP-1α
•    MIP-1β
•    PDGF-BB
•    RANTES
•    TNF-α
•    VEGF

Please mention if the cytokines/chemokines not appearing in the manuscript were undetectable (not very likely for e.g. MIP-1alpha/beta and CXCL10), or why they were not selected for analysis. I assume some of these quantifications were previously published (IFN-gamma, IL1beta, IL1RA,…), so it would be correct to state this as a reanalysis.

***You are right in stating that we have assayed 27 analytes. However, in this manuscript we included only cytokines and chemokines relevant to the study. We have already reported several cytokines/chemokines in our previous papers (DOI: 10.3390/pathogens10040498 PMID: 33924130; DOI: 10.1016/j.cyto.2019.154788 PMID: 31357078; DOI: 10.1093/infdis/jiab320 PMID: 34139757; DOI: 10.1093/cid/ciz810 PMID: 31722386).***

2. Statistical reporting of the results is incorrect and often misleading:
2.1. Odds ratios are interpreted as if they were relative risk (RR): RR = 1.74 means exposed individuals are 74% more likely to be diseased, OR = 1.74 means that the odds of disease is 74% higher in exposed individuals, which is intrinsically different. For instance, in line 412: “Here, we showed that the GG  genotype of the MIR499A rs3746444 is associated with a 74% increased risk of developing CL. Similarly, the G allele confers a 27% elevated risk of developing CL suggesting that the G allele may contribute to the development of Lg-CL.”

***We reported our results according to the interpretation of p-values statements from the American Statistical Association or e.g. the Nature editorial 20 March 2019: a low p-value just indicates a sufficient sample size, but does not indicate anything about the effect size or the 'strength' of the association.***

***However, we have corrected according to your suggestions.***

2.2. Large CI including 1 are often arguing against the significance claimed by the authors, e.g. “Carriers of rs2910164 CC genotype have a 33% elevated risk with a 95% confidence limits ranging from less 4% to an increase of 83%” should be reported as “no significantly increased odds of disease for the CC genotype.

***We have interpreted the odds ratios according to the American Statistical Association or e.g. the Nature editorial 20 March 2019. Following your comments, we described as suggestive the association with the C allele. Infectious diseases are complex and not one gene is involved in the development of the disease. Hence, we are interpreting the odds ratio just as suggestive.***

2.3. Raw p-values, uncorrected for age and gender are mentioned in the abstract, while there is a bias between cases and controls and none of the associations remain. As discussed below, the significant findings in males only also make more sense if occupational exposure is considered, this should be emphasized instead of the many weak associations.

Please correct the entire results section in the abstract accordingly: “Carriers of rs2910164 CC genotype have a 33% elevated risk while individuals with the genotype GG have lower risk of developing CL (OR=0.75 95%CI 0.55 - 1.0). Heterozygous GC individuals also have lower risk of developing CL compared with homozygous carriers of the C allele (OR= 0.71 95%CI 0.52 - 0.99 p=0.04). In addition, individuals homozygous for the G allele seem to have higher plasma IL-

8 and RANTES. For the MIR499A rs3746444, individuals with the GG genotype have a 74% increased risk of developing CL when compared to AA genotype. The G allele confers a 27% elevated risk of developing CL suggesting that the G allele may contribute to susceptibility to the development of Lg-CL (OR=1.27 (95% CI 1.0 – 1.6] p=68 0.035). Individuals homozygous for the G allele have higher plasma IL-8 with a tendency to also have high IL-6 and IL-17. Both MIR146A rs2910164 and MIR499A rs3746444 are associated with the development of Lg-CL and this association is more prevalent in male individuals.”

*We have corrected all the P values for cytokine/genotypes by False Discovery rates. We have also corrected all the p values adjusted for sex and age for comparison of genotypes.*
3. The paper does not have a “Limitations of the study” section. This should be inserted and at least include 1) the problem of multiple testing of a large number of associations, 2) the significant bias in both age and gender in cases vs controls, and 3) the assumption that all controls were exposed by merely living in the same endemic area might not hold, thus precluding a correct interpretation of the genetic associations. Other genetic studies have included DTH+ individuals which have been exposed to Leishmania In fact, the significant associations observed in males but not females might hint at different exposures by gender (mostly agricultural workers.

***We have included a paragraph line 502-509.***

Minor comments:

1. Several typos and spelling/grammatical errors are found throughout the manuscript.
   2. A (post-hoc) power calculation would be useful for the correct interpretation of the results, e.g. “with the available number of xx cases and xx controls and MAF of >0.15, an OR of xx (or larger) could be detected with 80% power.”

***Included in the section of Materials and Methods.***

Reviewer #3: Conclusions, as presented in the abstract, are not supported by the data (as detailed above) and should be rephrased, with an emphasis on the findings in males. A "limitations of the study" section is lacking. Public health relevance has been thoroughly addressed for cutaneous leishmaniasis.

***We have rephrased the discussion accordingly***.

Reviewer #3: A large sample size and correct design are the strengths of this study, while overselling and not correcting statistical findings are the main limitations.

***We appreciate your comments and have worked the discussion and correction of P values accordingly***
